# Outcomes of Systematic Transrectal Ultrasound-Guided Prostate Biopsy Performed by a Surgical Care Practitioner and Implications for Resource-Poor Countries

**DOI:** 10.7759/cureus.74488

**Published:** 2024-11-26

**Authors:** Reginald Ononye, Joanne Roberts, Kenechukwu Igbokwe, Ajibola A Adebisi, Mayowa Adefehinti

**Affiliations:** 1 Urological Surgery, Glan Clwyd Hospital, Bodelwyddan, GBR; 2 Trauma and Orthopaedics, Gateshead Health NHS Foundation Trust, Gateshead, GBR; 3 General Surgery, Epsom and St. Helier University NHS Foundation Trust, London, GBR; 4 Urology, Peterborough City Hospital, Peterborough, GBR

**Keywords:** prostate cancer, prostate cancer diagnosis, resource-poor countries, surgical care practitioner, systematic prostate biopsy, transrectal prostate biopsy

## Abstract

Introduction

Prostate cancer remains the most prevalent cancer among men and continues to present a significant public health challenge globally. The disease's growing prevalence has heightened the demand for skilled professionals capable of obtaining histological samples for accurate diagnosis, as tissue biopsy remains the cornerstone for diagnosing prostate cancer. Surgical care practitioners have become integral to the surgical team, and their roles have expanded to include performing biopsies. This paper evaluates the outcomes of transrectal ultrasound-guided (TRUS) prostate biopsies conducted by a surgical care practitioner (SCP) and explores the implications for resource-poor countries.

Methods

We retrospectively collated data from 218 patients who underwent TRUS prostate systematic biopsy by a surgical care practitioner between 2020 and 2022. We evaluated the prostate-specific antigen (PSA) values, MRI Likert score where available, and histological data and determined diagnostic yield and complication rates.

Results

The mean age and PSA level of the men were 69.7 years and 61.2 ng/ml, respectively; an average of 12 cores were obtained per biopsy. The cancer detection rate was 128/218 (59%), with a mean Gleason grade of 2.8. From available MRI, Likert 3 was the most common finding, 45/103 (43.6%), and prostate cancer was found in 40%. The mean MRI Likert scores for a positive and negative biopsy were 4 and 3.3, respectively. We recorded three complications (1%), all Clavien-Dindo 1 to 2, with no mortality.

Conclusion

A well-trained, supported, and supervised surgical care practitioner can safely and effectively perform TRUS systematic prostate biopsies and may improve access to prostate cancer diagnosis in developing countries.

## Introduction

With incidence rates steadily increasing, prostate cancer remains the most prevalent cancer among men and continues to pose a serious public health concern worldwide [1]. While clinical evaluation through laboratory tests, imaging, and other diagnostic methods is vital, tissue biopsy remains the cornerstone for diagnosing prostate cancer. The disease's growing prevalence has heightened the demand for skilled professionals capable of obtaining histological samples for accurate diagnosis [2]. The annual demand for prostate biopsies has risen significantly, surpassing two million procedures conducted via the transrectal (TR) route alone [3]. Advances in the understanding of prostate cancer biology and the development of new drugs and therapies have expanded treatment options and underscored the importance of early detection [4]. Over the years, biopsy techniques have evolved from blind, finger-guided transrectal core samples to ultrasound-guided procedures, transperineal (TP) approach, and, more recently, fusion technology incorporating advanced software [5]. The primary aim has been to improve the detection rates of clinically significant prostate cancer while minimising complications [5].

Surgical care practitioners (SCPs), introduced globally in the early 1990s, have gained recognition as vital members of surgical teams [6]. In the field of urology, their role has expanded to include performing minor surgical procedures under supervision, such as obtaining biopsy samples for histological analysis [7]. SCPs contribute to maintaining continuous patient care, particularly in response to medical workforce restructuring and compliance with the European working time directive [8]. One important area where they provide service is conducting prostate biopsies, aiding in the timely diagnosis of prostate cancer to support treatment and potentially enhance patient outcomes [7]. While TP prostate biopsy has largely replaced the TR approach in many developed nations, transrectal ultrasound-guided (TRUS) prostate biopsy remains the global standard [6]. Properly trained SCPs can improve access to prostate biopsies, ensuring that men in need receive prompt diagnostic services to initiate treatment [7]. This paper evaluates the outcomes of TRUS prostate biopsies conducted by a surgical care practitioner and explores the implications for resource-poor countries.

## Materials and methods

Prostate biopsy protocol

The study was carried out at Glan Clwyd Hospital, UK, using the established local prostate biopsy protocol adapted from the National Institute of Care and Excellence (NICE) guidelines [9]; all men with persistently raised prostate-specific antigen (PSA) or clinically abnormal feeling prostate on examination were offered prostate biopsy, only patients under 75 years (local age cut-off for radical treatment) were offered pre-biopsy multiparametric magnetic resonance scan of the prostate (mpMRI), reported by a consultant uro-radiologist (mpMRI has long been recognised as a useful tool to aid clinicians in determining the necessity of a biopsy, a higher Likert score generally suggests a greater suspicion of clinically significant prostate cancer, it also helps in treatment planning for radical treatment with curative intent). Patients with mpMRI Likert score of 3 or more were routinely offered a prostate biopsy. In the presence of a strong clinical indication (abnormal digital rectal exam, markedly raised PSA), a prostate biopsy was offered regardless of the mpMRI Likert score.

Patients were counselled on the TRUS and TP techniques within the context of the local service constraints and wait times. The choice of technique was guided by the following: location of lesions on mpMRI, previous biopsy status, and patient factors and choice (anterior lesions, previous negative TRUS prostate biopsy, and active surveillance were clinical indications for the TP route).

TRUS prostate biopsy was performed as a day case under a three-day antibiotic cover commencing 30 minutes before the procedure. Rectal antisepsis was standard in all patients using a 10% povidone-iodine wash to reduce the risk of infectious complications, as shown by Ergani et al. 2020 [10]. The Bk medical Flex 800^TM^ ultrasound (GE HealthCare, Chicago, IL) and Bard^TM^ biopsy gun were used to obtain the prostatic cores following a periprostatic block with 10 ml of 1% plain lidocaine. TP prostate biopsy is performed exclusively by urologists and is beyond the scope of this study. The prostatic cores were reviewed by a consultant pathologist and given a Gleason score for positive biopsies, otherwise reported as benign, to guide treatment decisions.

Data collection

Data from 218 consecutive biopsy-naïve men who underwent TRUS prostate biopsies performed independently by an SCP between January 2021 and December 2022 were retrospectively collected. The SCP had been trained to carry out standard systematic 12-core biopsies using the modified Gore technique, with supervision from a consultant urologist for the initial 100 procedures.

Demographic and clinical data, including age, PSA levels, MRI Likert scores (when available), and histological results, were compiled and analysed using an Excel spreadsheet. Basic statistical methods were used to calculate means and standard deviations for quantitative data. Yield and complication rates were determined from the histological data and compared with MRI Likert scores. Positive biopsy included all pathology reports where prostate cancer was detected regardless of the Gleason score.

## Results

The mean age of the study population was 69.8 years, ranging from 45 to 86 years. The average PSA value was 61.2 ng/ml, ranging from 0.8 to 4192 ng/ml. Each biopsy obtained an average of 12 cores. Pre-biopsy mpMRI prostate scans were conducted for 103 men (47% of the total population), with the average Likert score found to be 3. This group's most common Likert score was also 3, found in 43.6% (45 out of 103) of cases.

Histological analysis showed that prostate cancer was detected in 128 out of the 218 men, resulting in a cancer detection rate of 59%. Out of the 103 men who had mpMRI, 55 (53.5%) had prostate cancer regardless of the Likert score. The mean Likert scores for positive and negative (benign) histology were 4 and 3.3, respectively. The mean Gleason grade was 2.8 (Table 1).

**Table 1:** Summary of results ASAP: atypical small acinar proliferation; SD: standard deviation; N: number; PSA: prostate-specific antigen

Histology	Overall	Positive histology	Negative histology	ASAP
N (%)	218 (100%)	128 (59%)	87 (40%)	3 (1%)
Age (years)				
Mean ± SD	69.8 ± 8.0	70.8 ± 7.9	68.0 ± 8	-
Median (range)	71 (45 - 86)	72 (45 - 84)	69 (46 - 86)	-
PSA (ng/ml)				
Mean ± SD	57.4 ± 313.5	87.0 ± 402.6	8.8 ± 8.7	-
Median (range)	7.9 (0.8 - 4192)	9.4 (1.1 - 4192)	6.1 (0.8 - 56.8)	-
mpMRI				
N (%)	103 (47%)	55 (53.3%)	48 (46.6%)	-
Likert score mean ± SD	-	4.0 ± 0.9	3.3 ± 0.8	-
Gleason grade				
Mean ± SD	-	2.8 ± 1.5	-	-

**Figure 1:**
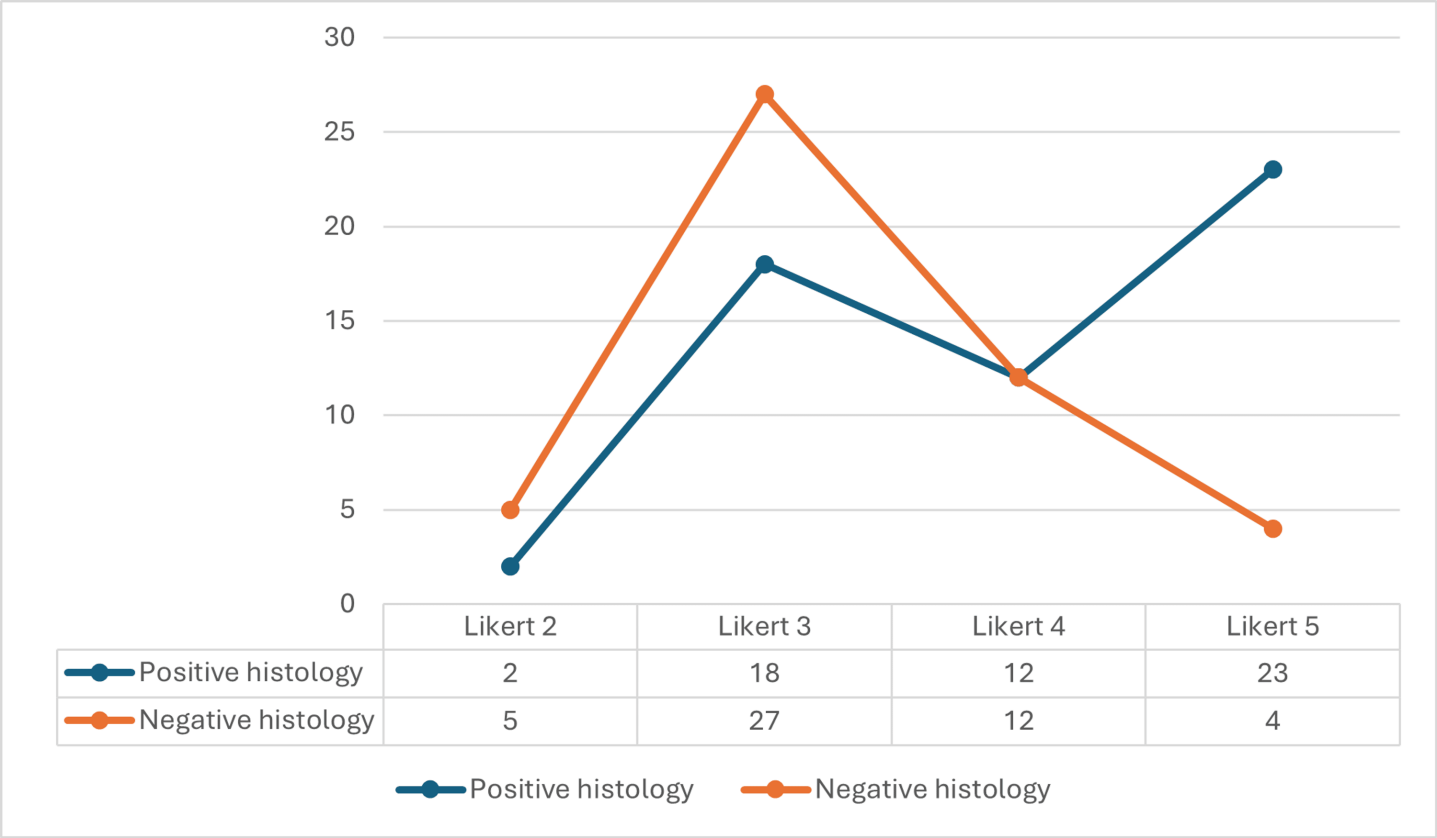
Distribution by Likert score and histology The MRI Likert score is a grading system used to evaluate prostate lesions on mpMRI. It ranges from 1 to 5: Score 1: highly unlikely to be cancerous; Score 2: unlikely to be cancerous; Score 3: indeterminate, uncertain for cancer; Score 4: likely cancerous; Score 5: highly likely cancerous

Prostate cancer was found in 85% of patients with mpMRI Likert score of 5, 50% with a score of 4, 40% with a score of 3, and 28% with a score of 2. There were no patients with an assigned Likert score of 1 (Figure 1). The study recorded three complications (1%), categorised within the Clavien-Dindo classification levels as 1 to 2 (vasovagal syncope in two patients and one patient developed a urinary tract infection requiring a full course of antibiotics). No mortality or hospital admissions for sepsis or haematuria were reported.

## Discussion

The global incidence of prostate cancer has been on the rise, with poorer outcomes disproportionately affecting resource-poor countries [11,12]. This has increased the need for skilled operators capable of obtaining prostate tissue for histological examination [13,14]. In the UK, the average waiting period for a prostate biopsy based on quality indices is 28 days, but there are patient reports of up to 70 days wait [15]. There is limited information on the waiting times for patients in resource-limited countries to receive a histological diagnosis and begin treatment after their initial hospital presentation; however, it is presumed to be longer, considering the significant shortage of trained urologists in these areas [16], as literature suggests that prostate biopsy is performed exclusively by urologists in resource-poor countries [17,18].

Due to the limited number of trainee surgeons, increased service demands, and shifts in work patterns, there is a growing need for surgical care practitioners (SCPs) to deliver healthcare services [8,19,20]. Globally, SCPs have been involved in various surgical tasks, from assisting in major procedures to independently performing less complex ones like biopsies [21].

Our study reported a TRUS prostate biopsy detection rate of 59%, which aligns with previously reported rates of 33-57% [22]. Additionally, we observed a low complication rate of 1%, indicating sufficient training and operator skill. This is consistent with the complication range of 0.1% to 7.0%, as documented by Liss et al. 2017 [23]. Moreover, our data indicated a 40% positivity rate for MRI Likert 3 lesions, higher than the 22.3% reported by Rosenkrantz et al. 2016 [24], suggesting operator proficiency. Higher mpMRI Likert scores were associated with a higher likelihood of detection of prostate cancer, with 55% of patients with a Likert of 3 and above diagnosed with prostate cancer (85% of Likert 5 scores), consistent with literature-reported trends [25]. Costa et al. in 2016 concluded that the Likert score was the strongest predictor of targeted biopsy positivity (O.R. 3.7, p < 0.001) [26].

The SCP in our study worked within a defined scope of practice, supported by a consultant, underscoring the importance of clear practice boundaries and implementation where adequate supervision, support, and governance are present; similar benefits are highlighted by Campaner 2019 [27]. Defining the scope of practice and standard operating procedures for SCP may pose a challenge [28], more so in settings of inadequate political commitment and weak regulatory oversight in resource-limited countries with implications for patient safety. It is critical to emphasise that SCPs should not replace the essential need for training and retaining surgeons in these regions.

There are notable limitations to our study. First, it was conducted at a single centre with procedures performed by one SCP, which may limit the generalisability of the results. Training, support, and supervision can differ between facilities and must be carefully evaluated when establishing similar services, especially in resource-limited settings. Second, the practice of prostate biopsy is evolving, with a shift from TRUS to the transperineal (TP) route in developed countries due to a lower risk of infection with TP biopsy [29]. While cancer detection rates are comparable, the TP approach is superior for targeting specific lesions and performing comprehensive template biopsies [29,30]. However, TP biopsies involve higher costs and a steeper learning curve, making widespread adoption in low-resource areas unlikely soon. Finally, this study utilised simple statistical methods without examining correlations or potential confounders.

## Conclusions

As demand for histological diagnosis of prostate cancer continues to surge, as well as increasing awareness and public health campaigns for early detection and treatment to improve outcomes, there will be increased pressure on surgical personnel who can deliver this service. There is a significant shortage of healthcare personnel and doctors worldwide, especially in developing countries. Our study adds to the existing evidence that a well-trained, supported, and supervised surgical care practitioner can safely and effectively perform TRUS prostate biopsies and may improve access to prostate cancer diagnosis in developing countries.
